# Smooth Muscle–Selective Inhibition of Nuclear Factor‐κB Attenuates Smooth Muscle Phenotypic Switching and Neointima Formation Following Vascular Injury

**DOI:** 10.1161/JAHA.113.000230

**Published:** 2013-06-21

**Authors:** Tadashi Yoshida, Maho Yamashita, Chihiro Horimai, Matsuhiko Hayashi

**Affiliations:** 1 Apheresis and Dialysis Center, School of Medicine, Keio University, Tokyo, Japan (T.Y., M.Y., C.H., M.H.)

**Keywords:** myocardin, Klf4, nuclear factor‐κB, smooth muscle cells

## Abstract

**Background:**

Vascular proliferative diseases such as atherosclerosis are inflammatory disorders involving multiple cell types including macrophages, lymphocytes, endothelial cells, and smooth muscle cells (SMCs). Although activation of the nuclear factor‐κB (NF‐κB) pathway in vessels has been shown to be critical for the progression of vascular diseases, the cell‐autonomous role of NF‐κB within SMCs has not been fully understood.

**Methods and Results:**

We generated SMC‐selective truncated IκB expressing (SM22α‐Cre/IκBΔN) mice, in which NF‐κB was inhibited selectively in SMCs, and analyzed their phenotype following carotid injury. Results showed that neointima formation was markedly reduced in SM22α‐Cre/IκBΔN mice after injury. Although vascular injury induced downregulation of expression of SMC differentiation markers and *myocardin*, a potent activator of SMC differentiation markers, repression of these markers and *myocardin* was attenuated in SM22α‐Cre/IκBΔN mice. Consistent with these findings, NF‐κB activation by interleukin‐1β (IL‐1β) decreased expression of SMC differentiation markers as well as *myocardin* in cultured SMCs. Inhibition of NF‐κB signaling by BAY 11‐7082 attenuated repressive effects of IL‐1β. Of interest, Krüppel‐like factor 4 (Klf4), a transcription factor critical for regulating SMC differentiation and proliferation, was also involved in IL‐1β‐mediated *myocardin* repression. Promoter analyses and chromatin immunoprecipitation assays revealed that NF‐κB repressed *myocardin* by binding to the *myocardin* promoter region in concert with Klf4.

**Conclusions:**

These results provide novel evidence that activation of the NF‐κB pathway cell‐autonomously mediates SMC phenotypic switching and contributes to neointima formation following vascular injury.

## Introduction

Vascular proliferative diseases including atherosclerosis and restenosis after percutaneous coronary interventions are the major source of death in westernized societies, being the underlying cause for subsequent complications such as ischemic heart disease and stroke. They are now recognized as an inflammatory disorder involving multiple cell types including endothelial cells, monocytes/macrophages, lymphocytes, neutrophils, and smooth muscle cells (SMCs).^1^ Elucidation of the molecular and cellular mechanisms underlying the progression of vascular diseases in each cell type is likely to provide novel a therapeutic approach to the diseases.

The nuclear factor‐κB (NF‐κB) family of transcription factors, particularly p65 and p50, participates in the pathogenesis of vascular proliferative diseases.^2^ In resting cells, NF‐κB exists in the cytoplasm as an inactive dimer by association with the inhibitory protein, Inhibitor of NF‐κB (IκB). On cell stimulation by inflammatory stress, IκB is phosphorylated on specific serine residues, serines 32 and 36, leading to its ubiquitination and consecutive proteasomal degradation. Released from IκB, NF‐κB is free to translocate into the nucleus, engage DNA, and initiate transcription of many genes including cytokines, chemokines, adhesion molecules, and antioxidant proteins. Results of previous studies showed that p65 and p50 were induced in the nuclei of vessels in response to arterial injury.^3–5^ Expression of NF‐κB was also detectable in the nuclei within human atherosclerotic lesions.^6–7^ Moreover, inhibition of the NF‐κB pathway reduced neointima formation following vascular injury.^8–13^ These results suggest that NF‐κB is a potential therapeutic target for vascular diseases including atherosclerosis. However, endothelium‐restricted inhibition of NF‐κB resulted in strongly reduced atherosclerotic plaque formation in *apolipoprotein E*–knockout mice fed a cholesterol‐rich diet.^14^ In addition, mice lacking the *p50* gene specifically within hematopoietic cells exhibited smaller atherosclerotic lesions in *low‐density lipoprotein receptor*–deficient mice.^15^ Myeloid‐specific deletion of IκB also resulted in larger and more advanced atherosclerotic lesions in these mice.^16^ As such, although results of the preceding studies provide compelling evidence that NF‐κB activation within endothelial cells and macrophages plays important roles for vascular proliferative diseases, the cell‐autonomous role of NF‐κB within SMCs in vivo remains to be determined.

During the formation of atherosclerotic lesions or in response to vascular injury, differentiated SMCs change their phenotype and enhance the activity of migration and proliferation.^1,17^ Because a hallmark of these changes in SMC phenotype is the downregulation of SMC differentiation markers including smooth muscle α‐actin (SM22α‐actin), SM22α, and SM myosin heavy chain (SMMHC), the underlying molecular mechanisms have been extensively studied in the last 2 decades.^17–19^ Most SMC differentiation maker genes contain common *cis* elements including multiple CC(A/T‐rich)_6_GG (CArG) elements and a transforming growth factor‐β (TGF‐β) control element in their promoter‐enhancer regions. The binding factor for CArG elements is serum response factor (SRF), which regulates expression of SMC differentiation marker genes by cooperating with its very strong coactivator, myocardin, or its corepressor, phosphorylated Elk‐1.^17–22^ Krüppel‐like factor 4 (Klf4) is a binding factor of the TGF‐β control element, and it potently represses SMC differentiation marker genes.^23–24^ Downregulation of SMC differentiation marker genes by platelet‐derived growth factor‐BB (PDGF‐BB) and oxidized phospholipids, both of which contribute to the formation of atherosclerosis, has been shown to be mediated through these *cis* elements and *trans*‐binding factors.^20,22–26^ Indeed, results of previous studies showed that PDGF‐BB and oxidized phospholipids, respectively, induced phosphorylation of Elk‐1 via activation of the MEK‐Erk1/2 pathway, and phosphorylated Elk‐1 competed with myocardin for SRF binding, resulting in the transcriptional repression of SMC differentiation marker genes.^20,22,25^ They also induced Klf4 expression, and small interfering RNA‐mediated knockdown of *Klf4* attenuated PDGF‐BB or oxidized phospholipid‐induced suppression of SMC differentiation marker genes in cultured SMCs.^24–26^ In addition, we demonstrated that conditional deletion of the *Klf4* gene in mice delayed repression of SMC differentiation markers and enhanced neointima formation following carotid injury in vivo.^27^ As such, studies thus far have indicated that Klf4 and phosphorylated Elk‐1 play critical roles in phenotypic switching of SMCs in response to atherogenic stimuli. However, the role of NF‐κB activation within SMCs in vivo for SMC phenotypic switching is unknown.

In the present study, we generated mice expressing a truncated form of IκB (IκBΔN) in a SMC‐selective manner by breeding mice expressing Cre recombinase under the control of the *SM22α* promoter (SM22α‐Cre mice)^28^ and IκBΔN mice.^29^ The IκBΔN mice contain the *IκBΔN* transgene separated from a universal CAG promoter by a floxed STOP sequence. Following the activation of Cre recombinase, they cell‐specifically express IκBΔN, which lacks its N‐terminal of 54 amino acids including 2 phosphorylation sites, thereby inhibiting NF‐κB activation. We examined the effects of SMC‐selective NF‐κB inhibition by IκBΔN on neointima formation following vascular injury. We also determined the mechanisms whereby NF‐κB activation induced SMC phenotypic switching by focusing on the transcriptional repression of the *myocardin* gene.

## Methods

### Generation of SMC‐Selective IκBΔN Transgenic Mice

Animal protocols were approved by Keio University Animal Care and Use Committee. IκBΔN mice were generated as previously described.^29^ SM22α‐Cre mice were provided by Dr Y. Eugene Chen (University of Michigan, Ann Arbor, MI).^28^ Heterozygous SM22α‐Cre mice were bred with heterozygous IκBΔN mice to generate SMC‐selective IκBΔN transgenic (SM22α‐Cre^+/−^/IκBΔN^+/−^; described as SM22α‐Cre/IκBΔN) mice and control (SM22α‐Cre^+/−^/IκBΔN^−/−^ or SM22α‐Cre^−/−^/IκBΔN^+/−^) mice. Both mice were mixed background strains of C57BL/6 and 129, and littermates were used for all comparisons. Genotyping was performed by PCR as described previously.^27,29^ Blood pressure and heart rate were measured by the tail‐cuff method (BP‐98E, Softron, Tokyo, Japan).

### Carotid Ligation Injury Model

Carotid artery ligation was performed as described previously.^27^ The right carotid artery was completely ligated just proximal to the carotid bifurcation. The left carotid artery served as an uninjured control. The right and left carotid arteries were harvested 3, 7, and 14 days after injury, fixed in 4% paraformaldehyde, and embedded into OCT compound. The arteries were also harvested for real‐time RT‐PCR and in vivo chromatin immunoprecipitation (ChIP) assays 3 days after injury.

### Morphometric Analysis

Cross‐sections of carotid arteries (6 μm) were prepared from 1.0 mm proximal to the ligature to the aortic arch. Morphometric analysis was performed using 3 sections per artery. These sections were located at ≈2.0 mm proximal to the ligature, and each section was 300 μm apart (ie, 1700, 2000, and 2300 μm proximal to the ligature). Sections were subjected to Verhoeff–van Gieson elastin staining, and the areas of the intima, media, and lumen measured by Image‐Pro Plus software (Media Cybernetics, Silver Spring, MD). Five mice for each genotype were analyzed.

### Immunohistochemistry

Immunohistochemistry was performed with antibodies for p65 (F6; Santa‐Cruz Biotechnology, Santa Cruz, CA), Ki67 (Santa Cruz Biotechnology), SM α‐actin (1A4; Sigma, St. Louis, MO), and SM22α (Abcam, Cambridge, MA). Staining for p65 and Ki67 was visualized by diaminobenzidine, and sections were counterstained by hematoxylin.^30^ Staining for SM α‐actin and SM22α was visualized by a Vector Red Alkaline Phosphatase Substrate kit (Vector Laboratories, Burlingame, CA), and sections were counterstained by hematoxylin.^27^ TUNEL staining was performed according to the manufacturer's instruction (Roche Diagnostics, Indianapolis, IN), and sections were counterstained by DAPI.^27^

### In Situ Hybridization

Tissue sections were permeabilized with proteinase K, treated with HCl, and acetylated with acetic anhydride in triethanolamine. Hybridization was performed using a digoxigenin‐labeled myocardin cRNA probe. Signals were detected by alkaline phosphatase–conjugated anti‐digoxigenin antibody (Roche Diagnostics). Sections were counterstained by nuclear fast red.

### Carotid Wire Injury Model

For morphometric analysis, a subset of mice was subjected to carotid wire injury as described previously.^31^ The injured and uninjured arteries were harvested 21 days after injury.

### Cell Culture

Rat aortic SMCs were cultured as described previously.^22,25,32^ Three days after plating at 10 000 cells/cm^2^, SMCs were treated with 5 ng/mL mouse interleukin‐1β (IL‐1β; R&D Systems, Minneapolis, MN). Treatment with BAY 11‐7082 (Calbiochem, Darmstadt, Germany) at 1 μmol/L or PD98059 (Cell Signaling Technology, Danvers, MA) at 10 μmol/L was performed 0.5 hours before IL‐1β treatment. Mouse aortic SMCs deficient for *Klf4* and control SMCs were described previously.^27^

### Real‐Time RT‐PCR

Total RNA prepared from the carotid arteries or cultured SMCs was used for real‐time RT‐PCR. Primer and probe sequences for *SM α‐actin*,* SM22α*,* SMMHC*,* myocardin*,* Klf4*,* Klf2*,* Klf5*, and *18S* rRNA were described previously.^24,32–34^ Primer sequences for *Vcam1*,* Icam1*, and *Ccl20* were as follows: *Vcam1*‐F—5′‐GCAAAGACAGGAGACATGGTA‐3′ and *Vcam1*‐R—5′‐TCACATCAAGTGTTAAACTTC‐3′; *Icam1*‐F—5′‐GTGGCGGGAAAGTTCCTG‐3′ and *Icam1*‐R—5′‐CGTCTGCAGGTCATCTTAGGAG‐3′; *Ccl20*‐F—5′‐TGGGTTTCACAACACAGATGG‐3′ and *Ccl20*‐R—5′‐CTTCTTGACTCTTAGGCTGAGG‐3′.

### Western Blotting and Immunofluorescence Studies

Western blotting and immunofluorescence studies were performed as described previously.^25,32^ Antibodies used were as follows: SM α‐actin (1A4), SM22α, p65 (F6), phospho‐p65 at serine 536 (93H1 Cell Signaling Technology), Erk1/2 (Cell Signaling Technology), phospho‐Erk1/2 (E10; Cell Signaling Technology), Elk‐1 (Santa‐Cruz Biotechnology), phospho‐Elk‐1 (Cell Signaling Technology), Klf4,^25^ IκB (Santa‐Cruz Biotechnology), GAPDH (6C5; Millipore, Billerica, MA), and FLAG (Sigma).

### Plasmid Constructs

An expression plasmid for IκBΔN was constructed by inserting a truncated IκB cDNA from amino acid 55 to amino acid 317 into a pCMV vector (Clontech, Mountain View, CA). The *myocardin* promoter‐luciferase construct and the *myocardin* enhancer‐promoter‐luciferase construct in a pGL2‐basic vector (Promega, Madison, WI) were kindly provided by Dr. Eric N. Olson (University of Texas Southwestern Medical Center, Dallas, TX),^35^ and they were cloned into a pGL3‐basic vector (Promega). Site‐directed mutagenesis of the *myocardin* promoter‐luciferase construct was performed using a QuikChange II site‐directed mutagenesis kit (Agilent Technologies, Santa Clara, CA).

### Transfection and Luciferase Assays

One day after plating at 10 000 cells/cm^2^, DNA plasmids were transfected into rat aortic SMCs using Superfect (Qiagen, Valencia, CA). On the next day, SMCs were treated with 5 ng/mL IL‐1β for an additional 24 hours. Luciferase activity was measured as previously described.^32^

### Quantitative ChIP Assays

Quantitative ChIP assays were performed using anti‐Klf4 antibody or anti‐p65 antibody as previously described.^25,33^ Real‐time PCR was performed to amplify the promoter region of the *myocardin* gene, which contains putative Klf4 binding sites and NF‐κB binding sites. Primer sequences for the *myocardin* promoter were as follows: Myoc‐proF—5′‐TGGGACCTTCATAAAGGCGTG‐3′ and Myoc‐proR—5′‐CCAGAAAACTGGCGCCTCC‐3′. Sequential ChIP assays were performed as described previously.^25^

### Statistical Analyses

**Figure 1.**
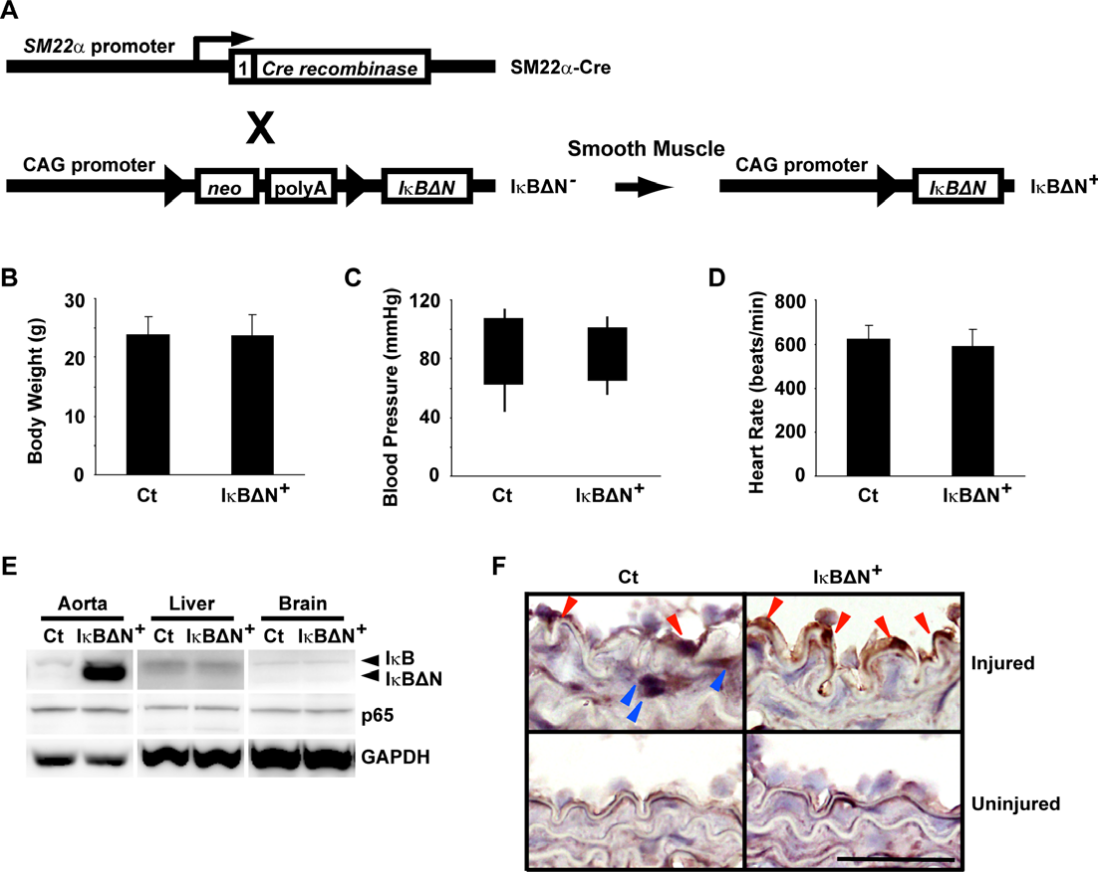
IκBΔN was predominantly expressed in the vessels of smooth muscle cell (SMC)–selective IκBΔN transgenic mice. A, Schematic representation of SMC‐selective expression of IκBΔN is shown. SM22α‐Cre mice were bred with IκBΔN mice to generate SM22α‐Cre/IκBΔN and control mice. Triangles represent the loxP sites. Number 1 represents exon 1 of the *SM22α* promoter; X, breeding; neo, *neomycin‐resistant* gene. B through D, Body weight (B, n=17 for each group), systolic and diastolic blood pressure (C, n=8 for each group), and heart rate (D, n=8 for each group) of SM22α‐Cre/IκBΔN (IκBΔN^+^) and control (Ct) mice were measured at 11 weeks of age. E, Expression of IκBΔN, IκB, p65, and GAPDH in the aortas, livers, and brains of SM22α‐Cre/IκBΔN and control mice was examined by Western blotting (n=4). F, Expression of p65 was examined by immunohistochemistry in the carotid arteries of SM22α‐Cre/IκBΔN and control mice on day 3 after ligation injury. Representative pictures are shown (n=4). Bar=50 μm. Red and blue arrowheads indicate p65‐positive endothelial cells and SMCs, respectively.

**Figure 2.**
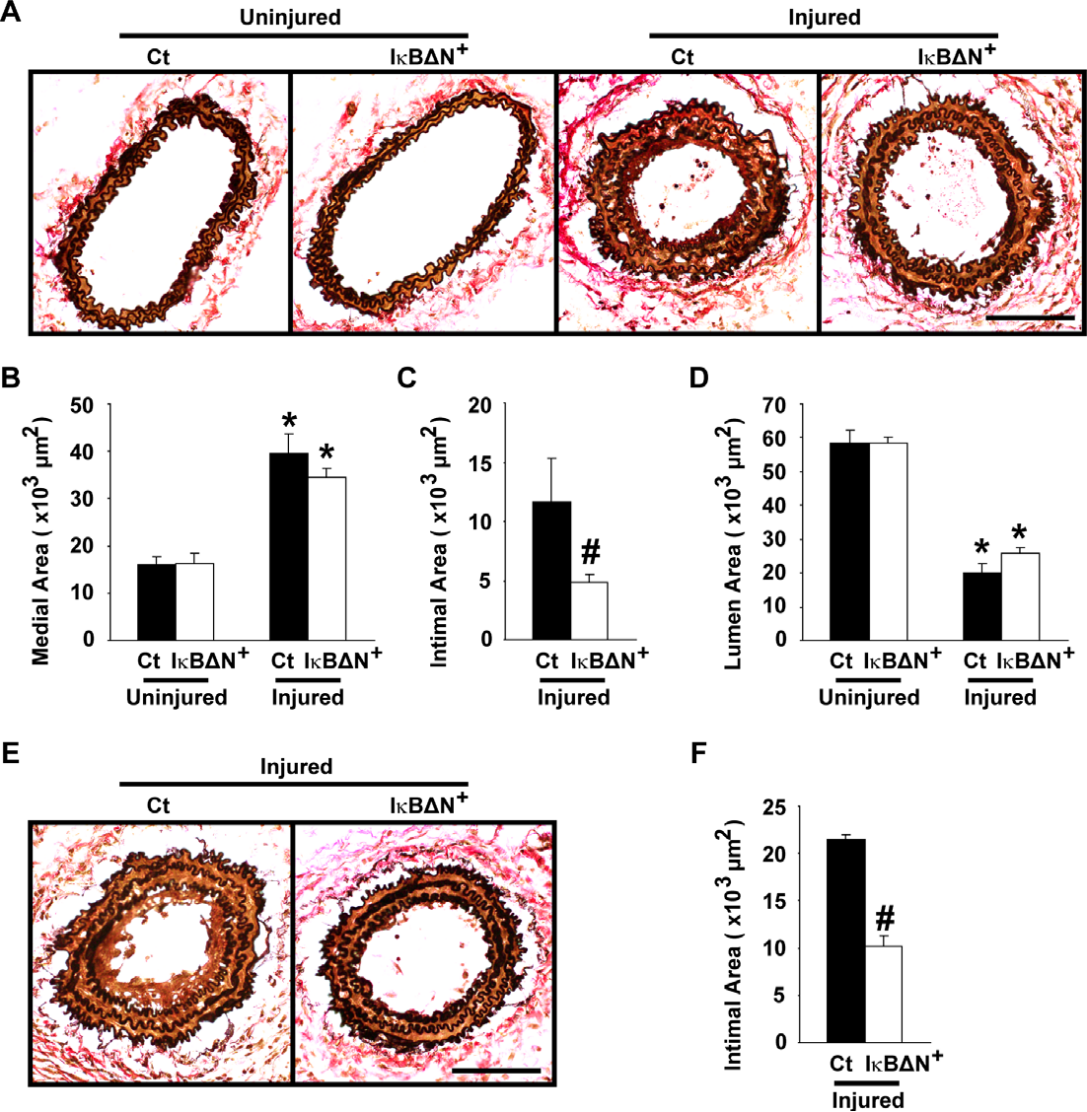
Neointima formation was reduced in smooth muscle cell (SMC)–selective IκBΔN transgenic mice following vascular injury. Verhoeff–van Gieson staining (A) was performed in the injured and uninjured carotid arteries of SM22α‐Cre/IκBΔN and control mice 14 days after ligation injury. Areas of the media (B), the intima (C), and the lumen (D) were quantified (n=5). Verhoeff–van Gieson staining (E) was performed in the injured and uninjured carotid arteries of SM22α‐Cre/IκBΔN and control mice 21 days after wire injury. The intima areas (F) were quantified (n=5). Bars=100 μm. *P<0.05 compared with uninjured arteries; #P<0.05 compared with corresponding control mice.

**Figure 3.**
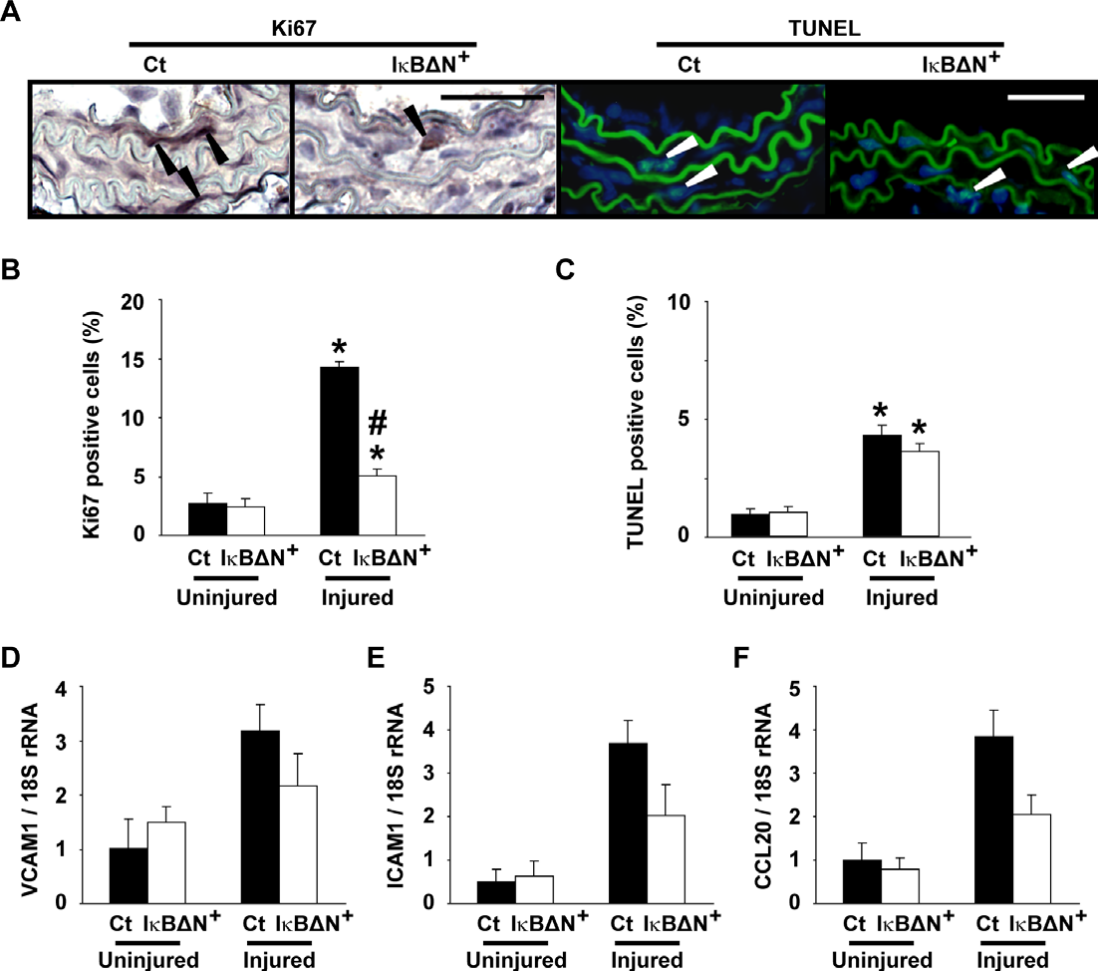
Injury‐induced smooth muscle cell (SMC) proliferation was attenuated in SMC‐selective IκBΔN transgenic mice. A, Representative pictures of injured carotid arteries for Ki67 staining on day 7 and TUNEL staining on day 3 after ligation injury in SM22α‐Cre/IκBΔN and control mice are shown. Bar=50 μm. Black and white arrowheads indicate Ki67‐ and TUNEL‐positive cells, respectively. Ratios of Ki67‐positive cells (B) and TUNEL‐positive cells (C) in the media were calculated (n=5). Expression of *Vcam1* (D), *Icam1* (E), and *Ccl20* (F) was determined by real‐time reverse‐transcription polymerase chain reaction (RT‐PCR) in the carotid arteries of SM22α‐Cre/IκBΔN and control mice on day 3 after ligation injury (n=4). TUNEL indicates terminal deoxynucleotidyl transferase dUTP nick end labeling; VCAM1, vascular cell adhesion molecule 1; ICAM1, intracellular cell adhesion molecule 1; CCL20, chemokine (C‐C motif) ligand 20. *P<0.05 compared with uninjured arteries; #P<0.05 compared with corresponding control mice.

Data are presented as mean±SEM except body weight, systolic and diastolic blood pressure, and heart rate, which are presented as mean±SD. Statistical analyses were performed by unpaired *t* tests (Figures 1B through 1D, 2C, 2F, 5B, and 6B), 1‐way factorial ANOVA (Figure 8A through 8C), 2‐way repeated‐measures ANOVA (Figure 5D), or 2‐way factorial ANOVA (Figures 2B, 2D, 3B through 3F, 4B through 4E, 5E, and 6C through 6E) with a post hoc Fisher protected least‐significant‐difference test. *P* values <0.05 were considered significant.

**Figure 4.**
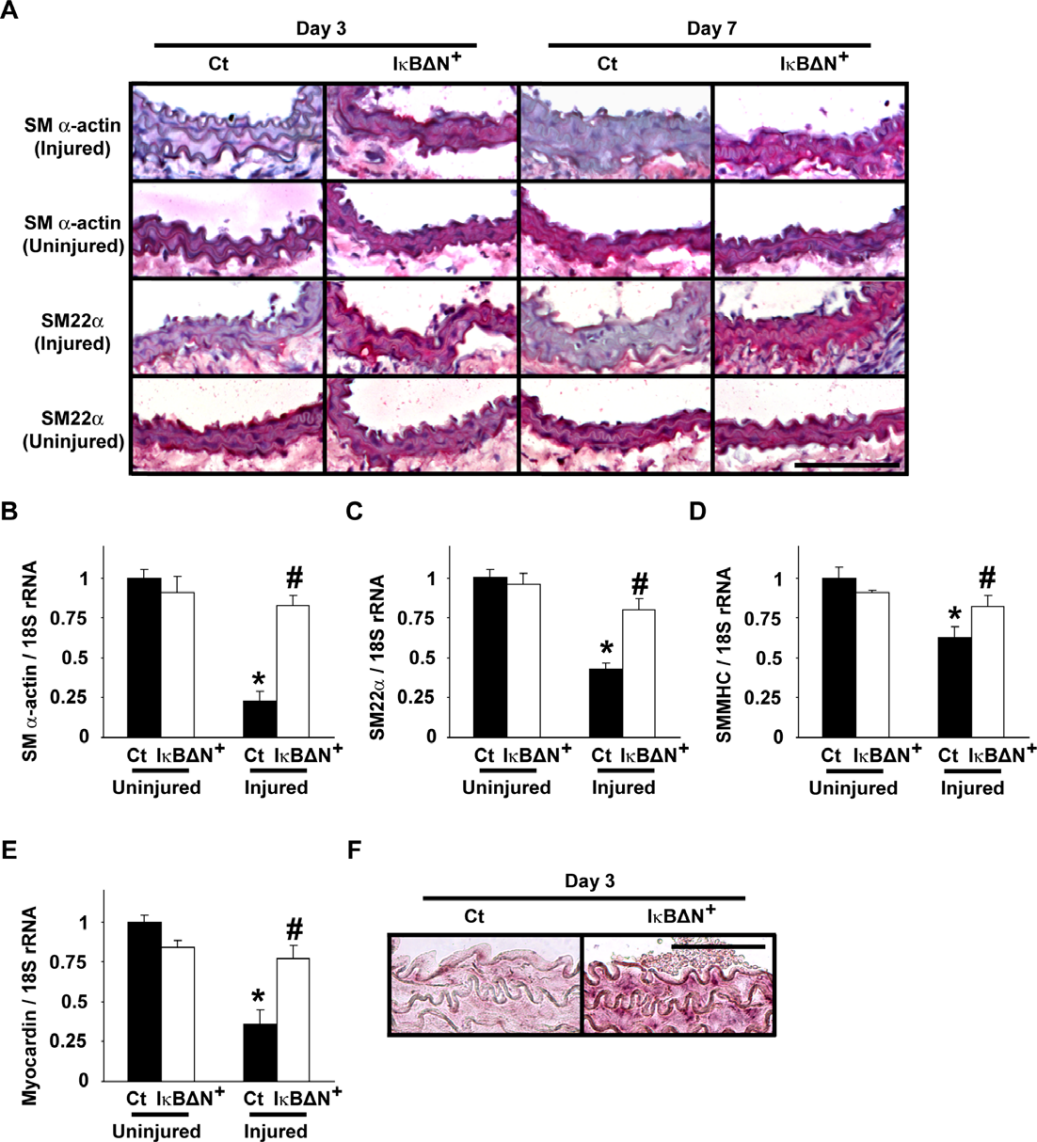
Injury‐induced repression of smooth muscle cell (SMC) differentiation markers and *myocardin* was attenuated in SMC‐selective IκBΔN transgenic mice. A, Expression of SM α‐actin and SM22α was examined by immunohistochemistry in the carotid arteries of SM22α‐Cre/IκBΔN and control mice on day 3 and day 7 after ligation injury. Representative pictures are shown (n=5). Bar=100 μm. Expression of *SM α‐actin* (B), *SM22α* (C), *SMMHC* (D), and *myocardin* (E) was determined by real‐time reverse‐transcription polymerase chain reaction (RT‐PCR) in the carotid arteries of SM22α‐Cre/IκBΔN and control mice on day 3 after ligation injury (n=4). *P<0.05 compared with uninjured arteries; #*P*<0.05 compared with corresponding control mice. F, Expression of *myocardin* was examined by in situ hybridization in the injured arteries on day 3 after ligation injury. Representative pictures are shown (n=4). Bar=50 μm.

**Figure 5.**
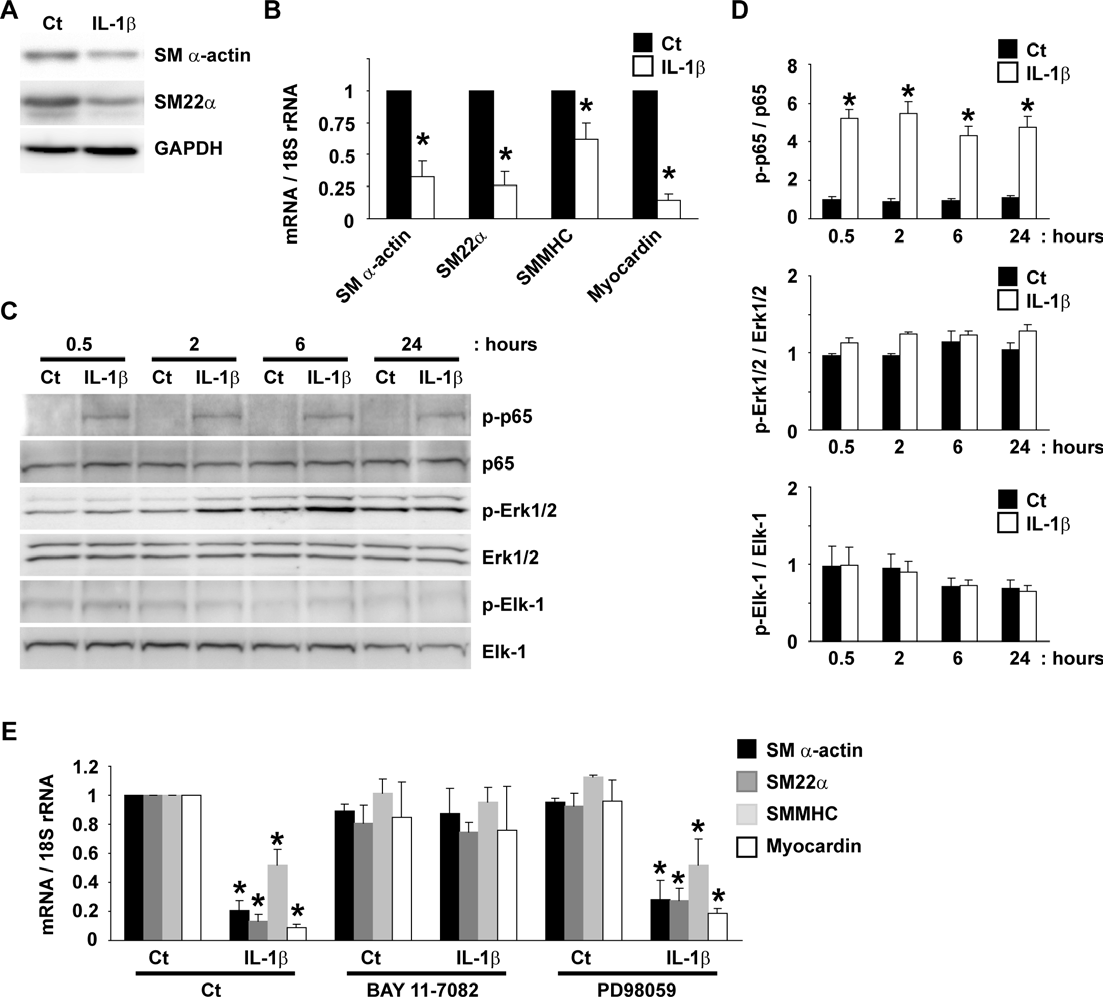
Interleukin 1β (IL‐1β) decreased expression of smooth muscle cell (SMC) differentiation markers and *myocardin* via activation of nuclear factor‐κB (NF‐κB). A and B, Cultured rat aortic SMCs were treated with 5 ng/mL IL‐1β or vehicle (Ct) for 24 hours. A, Expression of SM α‐actin, SM22α, and GAPDH was examined by Western blotting (n=4). B, Expression of SMC differentiation marker genes and *myocardin* was determined by real‐time reverse‐transcription polymerase chain reaction (RT‐PCR; n=4). C and D, Cultured SMCs were treated with IL‐1β or vehicle for indicated times, and expression of phosphorylated p65, p65, phosphorylated Erk1/2, Erk1/2, phosphorylated Elk‐1, and Elk‐1 was examined by Western blotting (n=4). D, Ratios of phosphorylated form to total form of p65, Erk1/2, and Elk‐1 were calculated. E, Cultured SMCs were treated with IL‐1β or vehicle for 24 hours in the presence or absence of BAY 11‐7082 or PD98059. Expression of SMC differentiation marker genes and *myocardin* was determined by real‐time RT‐PCR. *P<0.05 compared with control. SMMHC indicates smooth muscle myosin heavy chain.

**Figure 6.**
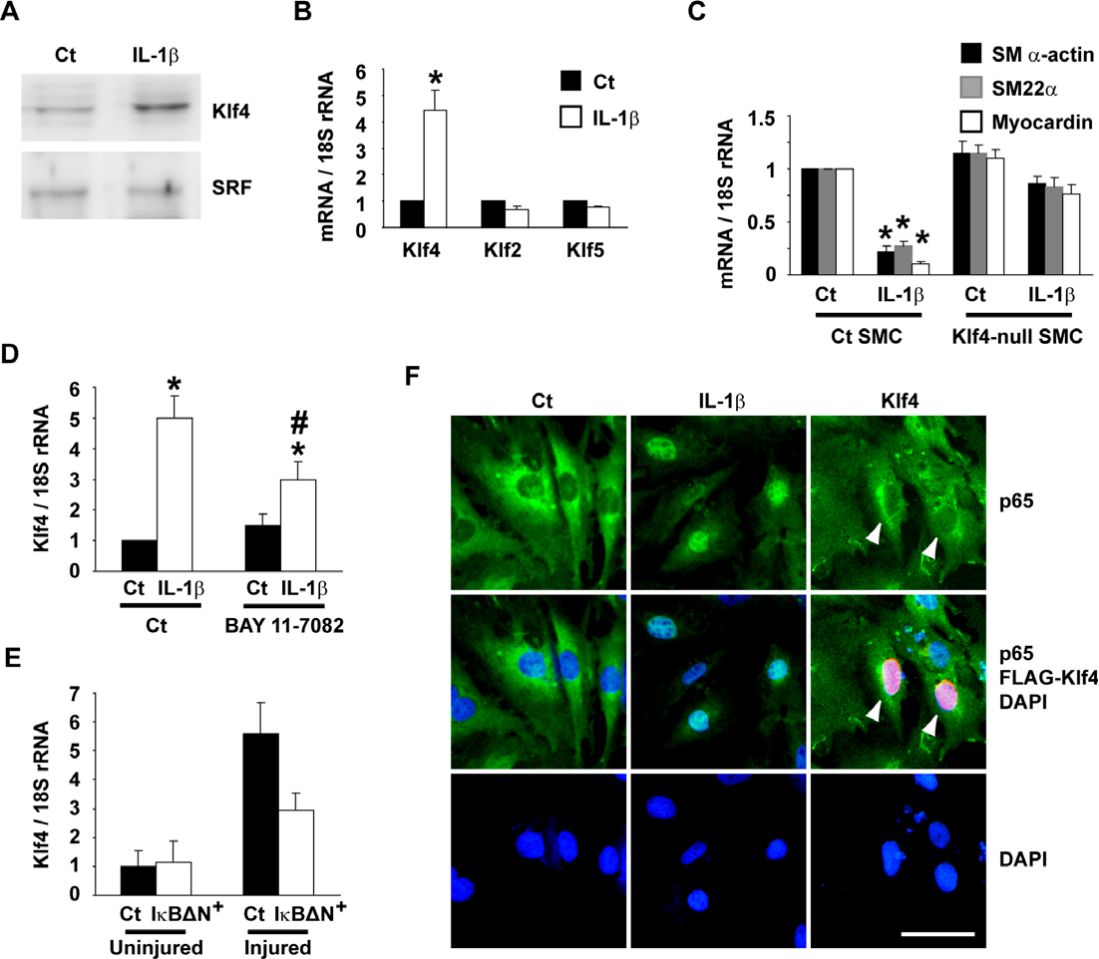
Interleukin 1β (IL‐1β)‐induced repression of smooth muscle cell (SMC) differentiation marker genes was mediated in part by Krüppel‐like factor 4 (Klf4). A and B, Cultured rat aortic SMCs were treated with IL‐1β or vehicle for 24 hours. A, Expression of Klf4 and serum response factor (SRF) was examined by Western blotting (n=4). B, Expression of *Klf4*,* Klf2*, and *Klf5* was determined by real‐time reverse‐transcription polymerase chain reaction (RT‐PCR; n=4). C, Expression of SMC differentiation marker genes and *myocardin* was determined by real‐time RT‐PCR in mouse *Klf4*‐deficient SMCs and control SMCs treated with IL‐1β or vehicle (n=4). D, Expression of *Klf4* was examined by real‐time RT‐PCR in rat aortic SMCs treated with IL‐1β or vehicle for 24 hours in the presence or absence of BAY 11‐7082 (n=4). E, Expression of *Klf4* was determined by real‐time RT‐PCR in the carotid arteries of SM22α‐Cre/IκBΔN and control mice on day 3 after ligation injury (n=4). F, Cultured rat aortic SMCs were treated with IL‐1β or vehicle or transfected with the expression plasmid for FLAG‐tagged Klf4. Top, intracellular localization of endogenous p65 (green) was examined by anti‐p65 antibody. Middle, merged images for endogenous p65 (green), FLAG‐tagged Klf4 (red), and DAPI nuclear staining (blue) are shown. Bottom, DAPI nuclear staining (blue) is shown. DAPI indicates 4', 6‐diamidino‐2‐phenylindole. Bar=50 μm. *P<0.05 compared with control; #*P*<0.05 compared with IL‐1β‐treated SMCs in the absence of BAY 11‐7082.

## Results

### SMC‐Selective Inhibition of the NF‐κB Pathway Reduced Neointima Formation Following Vascular Injury

Although activation of the NF‐κB pathway in vessels plays a key role for the progression of vascular diseases, the cell‐autonomous role of this pathway within SMCs has as yet been undetermined. We thus derived mice expressing the NF‐κB superrepressor IκBΔN in a SMC‐selective manner. Male heterozygous SM22α‐Cre mice were bred with female heterozygous IκBΔN mice to generate SM22α‐Cre/IκBΔN transgenic mice and control mice (Figure 1A). Transgenic mice were born at the expected Mendelian ratio and were grown to adults without any differences in visible appearance as compared with controls. Body weight (23.7±3.6 g in SM22α‐Cre/IκBΔN mice versus 23.9±3.0 g in control mice), systolic blood pressure (101.5±7.3 mm Hg in SM22α‐Cre/IκBΔN mice versus 107.5±7.0 mm Hg in control mice), diastolic blood pressure (64.4±7.2 mm Hg in SM22α‐Cre/IκBΔN mice versus 62.0±17.8 mm Hg in control mice), and heart rate (590±78 beats/minute in SM22α‐Cre/IκBΔN mice versus 624±64 beats/minute in control mice) at 11 weeks of age were similar in SM22α‐Cre/IκBΔN and control mice (Figure 1B through 1D). We confirmed overexpression of IκBΔN in the aortas, but not in the livers and brains, of transgenic mice (Figure 1E), indicating SMC‐selective Cre recombination in the mice. Endogenous expression of p65 and IκB in the aortas was unaffected by IκBΔN overexpression in SM22α‐Cre/IκBΔN mice (Figure 1E). SM22α‐Cre/IκBΔN mice and control mice at 12 to 13 weeks of age received carotid ligation injury. In uninjured arteries, p65 was faintly expressed in the cytoplasm in both SM22α‐Cre/IκBΔN and control mice (Figure 1F). Three days after injury, p65 was detected in the nuclei of endothelial cells and SMCs in the carotid arteries of control mice, whereas it was only seen in the nuclei of endothelial cells but not of SMCs in SM22α‐Cre/IκBΔN mice, suggesting that NF‐κB is selectively inhibited in SMCs in transgenic mice.

Morphometric analyses were performed in carotid arteries 14 days following ligation injury. Of major interest, SM22α‐Cre/IκBΔN mice exhibited decreased formation of neointima as compared with control mice (Figure 2A through 2D). Indeed, neointima area in SM22α‐Cre/IκBΔN mice (4883±696 μm^2^) was significantly smaller, by 42%, than that in control mice (11 649±3706 μm^2^) (Figure 2C). Medial areas of injured vessels were increased as compared with uninjured vessels in both SM22α‐Cre/IκBΔN and control mice, but not significantly different from one another (Figure 2B). Medial areas of uninjured vessels did not differ between SM22α‐Cre/IκBΔN and control mice. The lumen areas were slightly increased in SM22α‐Cre/IκBΔN mice (25 951±1689 μm^2^) compared with control mice (20 048±2627 μm^2^), maybe because of the reduced neointima formation in SM22α‐Cre/IκBΔN mice (Figure 2D). A subset of SM22α‐Cre/IκBΔN mice and control mice also received carotid wire injury. Neointima areas in SM22α‐Cre/IκBΔN mice (10 237±1022 μm^2^) were significantly smaller than in control mice (21 442±513 μm^2^) 21 days after wire injury (Figure 2E and 2F).

The rates of proliferation and apoptosis were determined in the media of vessels after ligation injury. Ki67 staining revealed that injured carotid arteries of SM22α‐Cre/IκBΔN mice (5.1±0.5%) exhibited significantly decreased proliferation compared with control mice (14.2±0.5%) on day 7 after injury, although the proliferation rate in injured vessels of both mice was increased compared with uninjured vessels (2.4±0.8% in SM22α‐Cre/IκBΔN mice and 2.8±0.9% in control mice; Figure 3A and 3B). In contrast, the apoptotic rate in the media did not differ between SM22α‐Cre/IκBΔN (3.6±0.4%) and control (4.3±0.5%) mice, although it was increased in injured vessels compared with uninjured vessels in both groups (1.0±0.3% in SM22α‐Cre/IκBΔN mice and 0.9±0.3% in control mice; Figure 3A and 3C). Expression of inflammation‐related genes was also determined by real‐time RT‐PCR. Although expression of *Vcam1*,* Icam1*, and *Ccl20* exhibited a tendency to be induced in the injured arteries of control mice, the induction was attenuated in SM22α‐Cre/IκBΔN mice (Figure 3D through 3F). The results were consistent with those of previous studies showing that inhibition of NF‐κB reduced the induction of inflammation‐related genes in response to IL‐1β or tumor necrosis factor‐α in cultured SMCs.^9,13,36–37^ Taken together, these results clearly demonstrated that inhibition of the NF‐κB pathway within SMCs attenuated the proliferation rate, the inflammatory response, and neointima formation following vascular injury and suggest that the decreased formation of neointima in transgenic mice is caused by reduced proliferation of medial cells, rather than by an altered apoptotic rate.

### Injury‐Induced Repression of Myocardin Expression Was Attenuated in SMC‐Selective IκBΔN Transgenic Mice

Because SMC phenotypic switching from differentiated SMCs to proliferating SMCs is triggered by the changes in expression of SMC differentiation markers, we examined whether SMC‐selective overexpression of IκBΔN affected downregulation of SMC differentiation markers following vascular injury. As shown in Figure 4A, expression of SM α‐actin and SM22α was markedly decreased in the medial layer of carotid arteries in control mice on day 3 and day 7 after ligation injury. However, of importance, injury‐induced repression of these SMC differentiation markers was not seen in carotid arteries of SM22α‐Cre/IκBΔN mice. Expression of *SM α‐actin*,* SM22α*, and *SMMHC* at mRNA levels was also decreased significantly in injured arteries of control mice, whereas the reduction was undetectable in SM22α‐Cre/IκBΔN mice (Figure 4B through 4D). Myocardin is a potent activator of these SMC differentiation markers.^18–19^ As shown in Figure 4E and 4F, *myocardin* mRNA expression was markedly decreased in injured arteries of control mice, but it was unaltered in SM22α‐Cre/IκBΔN mice, as determined by real‐time RT‐PCR and in situ hybridization. These results indicate that SMC‐selective inhibition of NF‐κB causes the attenuation of repression of SMC differentiation markers as well as *myocardin* in response to vascular injury. Results also suggest that the sustained expression of myocardin is likely to be responsible for the blunted repression of SMC differentiation markers in transgenic mice.

### IL‐1β Decreased SMC Differentiation Marker Genes and Myocardin Via NF‐κB Activation and Klf4 Induction

The NF‐κB pathway has been shown to be activated by IL‐1β treatment.^36^ To determine the mechanisms whereby NF‐κB activation within SMCs causes SMC phenotypic switching, cultured rat aortic SMCs were treated with IL‐1β. Instead of mouse SMCs, rat aortic SMCs were used for most of the in vitro experiments hereafter because: (1) sufficient amounts of mouse SMCs were unavailable because of the small volume of mouse aorta, (2) mouse SMCs grew up very slowly, and (3) there are many previous studies using rat aortic SMCs to investigate the mechanisms of SMC phenotypic switching.^9,13,20–27,20–34,36,38^ Treatment with IL‐1β for 24 hours decreased expression of SM α‐actin and SM22α in cultured SMCs (Figure 5A). Real‐time RT‐PCR analyses also showed that IL‐1β significantly decreased expression of multiple SMC differentiation marker genes including *SM α‐actin*,* SM22α*,* SMMHC*, and *myocardin* at mRNA levels (Figure 5B). Treatment with IL‐1β robustly induced expression of inflammatory markers *Ccl20* and *Vcam1* (data not shown), indicating that it did not elicit a global decrease in gene expression. Phosphorylation of p65 was induced 0.5, 2, 6, and 24 hours after IL‐1β treatment, whereas Erk1/2 phosphorylation was very modest (Figure 5C and 5D). Phosphorylation of Elk‐1 was not detectable by IL‐1β treatment at any time examined. Consistent with these findings, inhibition of the NF‐κB pathway by BAY 11‐7082 at 1 μmol/L abolished IL‐1β‐induced repression of SMC differentiation marker genes as well as *myocardin*, whereas PD98059, a MEK inhibitor, did not affect IL‐1β effects on these genes (Figure 5E). These results suggest that IL‐1β decreases expression of SMC differentiation marker genes as well as *myocardin* via activation of NF‐κB in cultured SMCs.

Klf4 has been shown to be involved in repression of SMC differentiation markers in response to PDGF‐BB and oxidized phospholipids.^24–26^ We examined the effects of IL‐1β on Klf4 expression. Of interest, Klf4 at both mRNA and protein levels was increased by IL‐1β in cultured SMCs, whereas other Klf family members, *Klf2* and *Klf5*, were not induced (Figure 6A and 6B). IL‐1β‐induced repression of SMC differentiation marker genes and *myocardin* was markedly attenuated in *Klf4*‐deficient SMCs (Figure 6C), suggesting that Klf4 also contributes to repression of these genes by IL‐1β. Compared with a 5‐fold induction of *Klf4* expression in the absence of BAY 11‐7082, IL‐1β induced *Klf4* expression only by 3‐fold in the presence of BAY 11‐7082 in cultured rat aortic SMCs (Figure 6D). Results suggest that IL‐1β‐induced *Klf4* expression is mediated, at least in part, by NF‐κB activation. Involvement of NF‐κB activation in *Klf4* induction was also seen in mice, in that the induction of *Klf4* expression was reduced in SM22α‐Cre/IκBΔN mice compared with control mice (Figure 6E). On the contrary, overexpression of Klf4 did not cause the translocation of p65 from the cytoplasm to the nucleus (Figure 6F), indicating that NF‐κB activation is not a downstream target of Klf4. Taken together, these results suggest that IL‐1β represses expression of SMC differentiation marker genes and *myocardin* in cultured SMCs via activation of the NF‐κB pathway and that the repressive effect of NF‐κB on these genes is partly mediated by Klf4 induction.

### NF‐κB and Klf4 Cooperatively Repressed Transcriptional Activity of the Myocardin Gene in Response to IL‐1β Treatment and Vascular Injury

**Figure 7.**
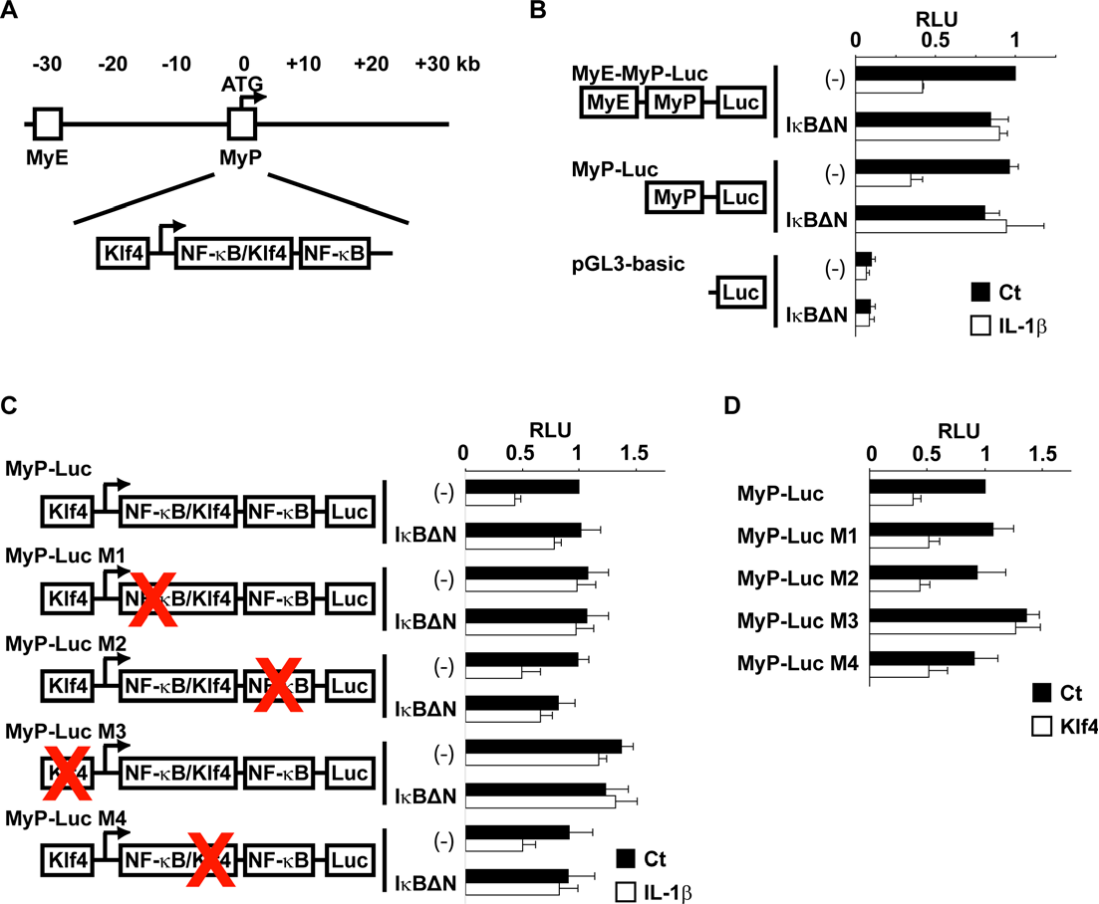
Interleukin 1β (IL‐1β) repressed the transcriptional activity of the *myocardin* gene through the consensus binding sites for nuclear factor‐κB (NF‐κB) and Krüppel‐like factor 4 (Klf4). A, Schematic representation of the *myocardin* gene is shown. The *myocardin* enhancer (MyE) is located ≈30 kb upstream from the transcriptional start site of the *myocardin* gene. The *myocardin* promoter (MyP), from −407 to +229 bp, contains a consensus Klf4 binding site, a hybrid sequence for the consensus Klf4 binding site and consensus NF‐κB binding site, and a consensus NF‐κB binding site. B, Cultured aortic smooth muscle cells (SMCs) were transfected with the *myocardin* enhancer‐promoter‐luciferase construct, the *myocardin* promoter‐luciferase construct, or a pGL3‐basic plasmid with the expression plasmid for IκBΔN. One day after transfection, SMCs were treated with IL‐1β or vehicle for an additional 24 hours. C, SMCs were transfected with mutational constructs of the *myocardin* promoter‐luciferase construct with the expression plasmid for IκBΔN. One day after transfection, SMCs were treated with IL‐1β or vehicle for an additional 24 hours. D, SMCs were transfected with the *myocardin* promoter‐luciferase construct or its mutants with the Klf4 expression plasmid. Luciferase activity was measured and normalized to protein content (n=4).

To clarify the mechanisms whereby IL‐1β represses *myocardin* expression via NF‐κB activation and Klf4 induction, the promoter‐enhancer activity of the *myocardin* gene was examined. Results of previous studies showed that the *myocardin* enhancer, which is a 350‐bp fragment located about 30 kb upstream of the transcriptional start site of the *myocardin* gene, was sufficient to control *myocardin* expression in the heart and SMCs during mouse embryogenesis and adulthood.^35^ This enhancer contains multiple *cis*‐regulatory elements including a Mef2 binding site, 5 Foxo binding sites, and a Tead binding site. We examined the effects of IL‐1β on the *myocardin* enhancer‐promoter‐luciferase construct, in which the 350‐bp *myocardin* enhancer was fused to the *myocardin* promoter (−407 to +229 bp) as well as to the *myocardin* promoter‐luciferase construct in cultured SMCs (Figure 7A). Results showed that IL‐1β repressed the transcriptional activity of the *myocardin* enhancer‐promoter‐luciferase construct and the *myocardin* promoter‐luciferase construct by 35% and 42%, respectively, and the effects were abolished by the presence of IκBΔN (Figure 7B). These results suggest that IL‐1β decreases the transcriptional activity of *myocardin* through the *myocardin* promoter, but not the *myocardin* enhancer, via NF‐κB activation. We found 2 consensus NF‐κB binding sites, 5′‐GG(G/A)(G/A)NN(C/T)(C/G)CC‐3′, at +113/+122 and +168/+177 bp, in addition to 2 consensus Klf4 binding sequences, 5′‐(G/A)(G/A)GG(C/T)G(C/T)‐3′, at −29/−23 and +115/+121 bp within the *myocardin* promoter. One of the consensus NF‐κB binding sites at +113/+122 bp contained a consensus Klf4 binding sequence at +115/+121 bp. Of these *cis* elements, mutational analyses revealed that mutation of the consensus NF‐κB binding site at +113/+122 bp and mutation of the consensus Klf4 binding site at −29/−23 bp abolished IL‐1β‐mediated repression of *myocardin* promoter activity in cultured SMCs (Figure 7C). Moreover, although overexpression of Klf4 decreased the transcriptional activity of the *myocardin* promoter, mutation of the consensus Klf4 binding site at −29/−23 bp blunted the Klf4 effect (Figure 7D). Results suggest that IL‐1β represses the transcriptional activity of the *myocardin* gene through 2 mechanisms: (1) activated NF‐κB binds to the consensus NF‐κB binding site at +113/+122 bp of the promoter; and (2) Klf4, which is induced in part by NF‐κB activation, binds to the consensus Klf4 binding site at −29/−23 bp of the promoter.

**Figure 8.**
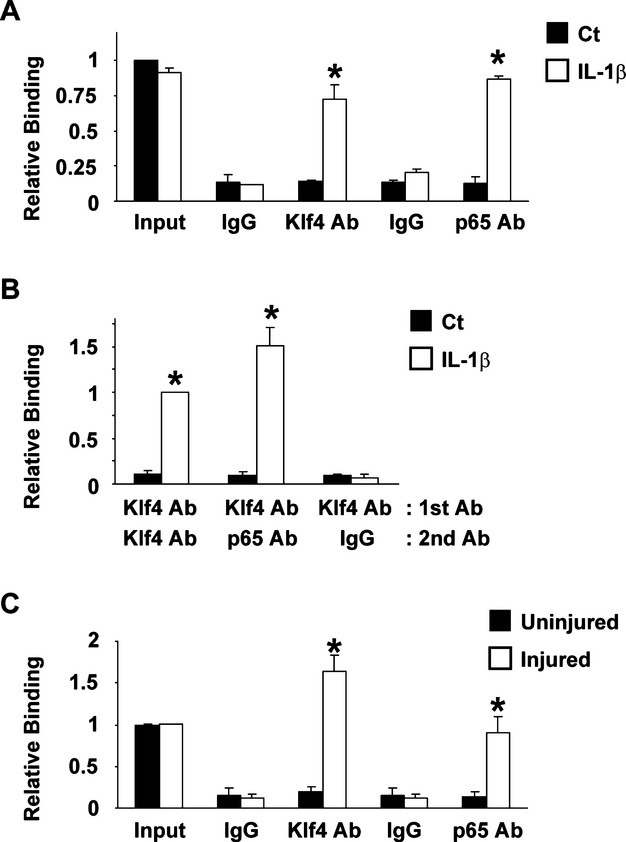
Both p65 and Krüppel‐like factor 4 (Klf4) bound to the *myocardin* promoter in cultured smooth muscle cells (SMCs) after interleukin 1β (IL‐1β) treatment and in the injured carotid arteries. A, Association of Klf4 and p65 with the *myocardin* promoter was determined by chromatin immunoprecipitation (ChIP) assays in cultured SMCs treated with IL‐1β or vehicle (n=3). B, Sequential ChIP assays were performed to test the cooperative binding of Klf4 and p65 to the *myocardin* promoter in cultured SMCs after IL‐1β treatment (n=3). C, Association of Klf4 and p65 with the *myocardin* promoter was examined by in vivo ChIP assays in the carotid arteries 3 days after ligation injury (n=3). *P<0.05 compared with control. IgG indicates immunoglobulin G.

Finally, quantitative ChIP assays were performed to determine if NF‐κB and Klf4 bound to the *myocardin* promoter. Results showed that both p65, an active component of NF‐κB, and Klf4 bound to the promoter region of the *myocardin* gene in cultured SMCs treated with IL‐1β (Figure 8A). Moreover, sequential ChIP assays showed that both factors bound to the same chromatin (Figure 8B). Furthermore, binding of p65 and Klf4 was also detectable at the *myocardin* promoter in the mouse carotid arteries 3 days after vascular injury (Figure 8C). These findings were consistent with results of the present (Figure 1F) and previous studies showing that both p65 and Klf4 were induced in the nuclei of arteries following vascular injury.^4–5,27^ Taken together, these results suggest that NF‐κB and Klf4 cooperatively represses the transcription of the *myocardin* gene within SMCs in response to IL‐1β treatment in vitro as well as vascular injury in vivo.

## Discussion

In the present study, we found that SMC‐selective expression of IκBΔN, the NF‐κB super‐repressor, in mice resulted in sustained expression of SMC differentiation markers and *myocardin* in carotid arteries after vascular injury. We also showed that injury‐induced increases in the proliferation rate of medial cells was blunted in SMC‐selective IκBΔN transgenic mice. As a result, neointima formation was significantly decreased in transgenic mice. Moreover, we demonstrated a novel mechanism whereby NF‐κB activation decreased *myocardin* expression by the binding of NF‐κB to the *myocardin* promoter region in concert with Klf4. Cooperative binding of NF‐κB and Klf4 was detectable in the carotid arteries after injury in vivo as well as in cultured SMCs treated with IL‐1β. As such, results of the present study provide clear evidence showing that NF‐κB activation within SMCs plays a critical role in SMC phenotypic switching and neointima formation following vascular injury.

Because NF‐κB has been considered a potential therapeutic target of vascular diseases, many studies were performed to examine the effects of NF‐κB inhibition on neointima formation following vascular injury. For example, adenovirus‐mediated transfer of IκB super‐repressor inhibited development of intimal hyperplasia after vascular injury in rats in vivo.^9^ Likewise, double‐stranded decoy oligonucleotides that bind NF‐κB and keep it localized in the cytoplasm decreased injury‐induced neointima formation in rats and pigs,^10–11^ as well as in‐stent restenosis in hypercholesterolemic rabbits.^12^ Moreover, antisense oligonucleotides that decrease p65 synthesis reduced neointima formation following carotid injury in rats.^8^ Most recently, the NF‐κB essential modulator‐binding domain peptide, which can block the activation of the IκB kinase complex and therefore inhibit NF‐κB activation, was also able to reduce injury‐induced neointima formation in rats and in *apolipoprotein E*–deficient mice.^13^ Although results of these studies suggest that NF‐κB inhibition is an effective therapeutic approach for vascular diseases, the target cell types had been unclear because of the global inhibition of NF‐κB activity in these studies. In this regard, results of the present study provide compelling evidence that NF‐κB activation within SMCs is critical for injury‐induced SMC phenotypic switching and neointima formation, although they do not deny a possibility that paracrine factors secreted by endothelial cells and/or monocytes/macrophages also affect the characteristics of SMCs. In fact, NF‐κB inhibition in endothelial cells and macrophages has also been shown to decrease the formation of atherosclerosis.^14–16^ Probably, NF‐κB activation in multiple cell types including SMCs would simultaneously enhance lesion formation.

Although the present study was focused on the mechanisms whereby NF‐κB activation decreased expression of SMC differentiation markers and *myocardin*, the reduced neointima formation is likely to be caused by the decreased proliferation rate in SMC‐selective IκBΔN transgenic mice. In support of this, constitutive expression of NF‐κB has been shown to be necessary for SMC proliferation in cultured bovine SMCs.^39^ Indeed, microinjection of either purified IκB or double‐stranded oligonucleotides harboring NF‐κB binding elements inhibited SMC proliferation in vitro. Regarding the underlying mechanisms, Zuckerbraun et al^9^ showed that the antiproliferative activity of IκB was related to cell‐cycle arrest through upregulation of the cyclin‐dependent kinase inhibitors p21^WAF1/Cip1^ and p27^Kip1^ in cultured SMCs. Moreover, of interest, myocardin has been shown to suppress SMC proliferation by inhibiting NF‐κB‐dependent cell‐cycle progression in cultured SMCs.^40^ Although the aforementioned mechanistic studies were mostly performed in cultured SMCs, it is highly possible that the decreased proliferation rate in our transgenic mice was also caused by these mechanisms.

A caveat in the present study is that expression of IκBΔN was also detectable in the heart (data not shown), because SM22α‐Cre mice were used in the study and SM22α is transiently expressed in the embryonic heart.^19^ However, blood pressure, heart rate, and changes in body weight in SMC‐selective IκBΔN transgenic mice were quite similar to control mice, suggesting that cardiac function of the transgenic mice was not impaired, and therefore the phenotype seen in the carotid arteries was mainly a result of vascular expression of IκBΔN. At present, most SMC differentiation markers are known to be transiently expressed in the heart during embryonic development.^18–19^ Nevertheless, it would be of interest to test the effects of SMC‐specific but not SMC‐selective inhibition of NF‐κB activation on the development of vascular disease, if SMC‐specific Cre recombinase expressing mice are developed in future.

Results of our present study showed that IL‐1β‐induced repression of SMC differentiation markers and *myocardin* was mediated in part by Klf4. In response to IL‐1β treatment, Klf4 and p65 bound to the promoter region of the *myocardin* gene within the same chromatin in cultured SMCs. Binding of these factors to the *myocardin* promoter was also seen in the carotid arteries following vascular injury. These results suggest that Klf4 contributes to phenotypic switching of differentiated SMCs into the inflammatory state of SMCs by cooperating with p65. However, the effect of Klf4 on SMC proliferation has been shown to be opposite from p65. Although NF‐κB enhances SMC proliferation, as described above, Klf4 has been shown to repress SMC proliferation by increasing p21^WAF1/Cip1^ expression via recruitment of p53 to the enhancer region of the *p21*^*WAF1/Cip1*^ gene in SMCs.^27,38^ As such, it is of interest to note that Klf4 and p65 cooperatively repress SMC differentiation marker genes, but they counteract each other for cellular proliferation.

In summary, we have provided novel evidence that NF‐κB activation within SMCs causes SMC phenotypic switching and neointima formation in concert with Klf4. NF‐κB inhibitors exhibiting an affinity for SMCs would be a candidate for treatment of vascular diseases including atherosclerosis.
